# Multi-mode humidity sensing with water-soluble copper phthalocyanine for increased sensitivity and dynamic range

**DOI:** 10.1038/s41598-017-10401-2

**Published:** 2017-08-30

**Authors:** Eric S. Muckley, Christopher B. Jacobs, Keith Vidal, Nickolay V. Lavrik, Bobby G. Sumpter, Ilia N. Ivanov

**Affiliations:** 1 Center for Nanophase Materials Sciences, Oak Ridge National Laboratory, P.O. Box 2008, Oak Ridge, TN 37831-6496 USA; 2 Bredesen Center, University of Tennessee Knoxville, 444 Greve Hall, 821 Volunteer Blvd, Knoxville, TN 37996-3394 USA; 3 Computer Science and Mathematics Division, Oak Ridge National Laboratory, Oak Ridge, TN 37831 USA

## Abstract

Aqueous solubility of copper phthalocyanine-3,4′,4″,4″′-tetrasulfonic acid tetrasodium salt (CuPcTs) enables fabrication of flexible electronic devices by low cost inkjet printing. We (1) investigate water adsorption kinetics on CuPcTs for better understanding the effects of relative humidity (RH) on hydrophilic phthalocyanines, and (2) assess CuPcTs as a humidity-sensing material. Reaction models show that H_2_O undergoes 2-site adsorption which can be represented by a pair of sequentially-occurring pseudo-first order reactions. Using high frequency (300–700 THz) and low frequency (1–8 MHz) dielectric spectroscopy combined with gravimetric measurements and principal component analysis, we observe that significant opto-electrical changes in CuPcTs occur at RH ≈ 60%. The results suggest that rapid H_2_O adsorption takes place at hydrophilic sulfonyl/salt groups on domain surfaces at low RH, while slow adsorption and diffusion of H_2_O into CuPcTs crystallites leads to a mixed CuPcTs-H_2_O phase at RH > 60%, resulting in high frequency dielectric screening of the film by water and dissociation of Na^+^ from CuPc(SO_3_^−^)_4_ ions. The CuPcTs-H_2_O interaction can be tracked using a combination of gravimetric, optical, and electrical sensing modes, enabling accurate ( ± 2.5%) sensing in the ~0–95% RH range with a detection limit of less than 0.1% RH.

## Introduction

Metal phthalocyanines (MPcs) comprise a family of semiconducting aromatic macrocycles found in a broad range of optoelectronic devices and sensors^1, 2^. Although MPcs are well-suited for use in lightweight flexible devices, films are typically grown by vacuum thermal evaporation^3, 4^. Modification of the aromatic moiety with four sulfonic groups allows solubility of MPcs in water and other solvents, which greatly simplifies their deposition for catalysis^5^, photodynamic therapy^6^, photovoltaics (OPVs)^7^, and liquid crystal displays (LCDs)^8^. Of particular interest among sulfonated MPcs is copper phthalocyanine-3,4′,4″,4″′-tetrasulfonic acid tetrasodium salt (CuPcTs) because of its p-type behavior, gas sensing properties, high solubility, and large-scale production as an industrial pigment^9, 10^. Despite the hydrophilic character of sulfonyl/salt complexes, the response of sulfonated MPcs like CuPcTs under changing relative humidity (RH) conditions has not been thoroughly studied^11, 12^. Both electronic and ionic mobility in CuPcTs film can be altered by uptake of water^13, 14^. The presence of H_2_O at SO_3_Na groups induces salt dissociation, leading to formation of Na^+^ and CuPc(SO_3_^−^)_4_ ions^15, 16^ which contribute to charge transport in CuPcTs at RH > 45%^12^. Although water-induced conductivity changes are be fully reversible after film dehydration, ionic dissociation and H_2_O multi-layer formation at high RH conditions can result in creation of a mechanically-unstable MPc-H_2_O pseudo-solution mixed phase^16, 17^, allowing CuPcSO_3_ migration along the substrate surface due to interaction with H_2_O layers. Mobility of CuPc(SO_3_^−^)_4_ ions can result in morphological changes including domain displacement and fracture^11, 18^.

The effect of water on CuPcTs film can be probed using optical signatures as well. While optical response of sulfonated phthalocyanines has been well-studied in solution, less information is available about water interaction with solid films^15, 19^. The electronic absorption spectrum of CuPc has two prominent features which arise from π → π^*^ transitions: the Q band (~600–750 nm), associated with a_1u_ to e_g_^*^ orbital transitions, and the B (Soret) band (~300–450 nm), associated with a_2u_ to e_g_^*^ orbital transitions^20, 21^. In crystals with unit cells composed of *n* molecules, the nondegenerate excited states of each molecule are comprised of *n* bands, resulting in several absorption peaks^22^. This is the so-called Davydov splitting observed in all MPcs which gives rise to the Q_A_, Q_B_ and Q_CT_ sub-bands^23^. Changes in the optical spectra are related to specific electronic transitions which reveal the effect of water on charge transfer (Q_CT_), excitonic transitions (Q_A_, and Q_B_), and bandgap energy. While Ghani *et al*. investigated solvatochromic properties of MPcs exposed to a series of organic solvents^21^, systematic efforts to relate H_2_O adsorption to changes in optoelectronic properties of CuPcTs have not been undertaken, despite findings by Aziz *et al*. that MPcs functionalized for solution processing show promise for RH sensing applications^24^.

Here we employ multi-modal probing of the CuPcTs response to water vapor by connecting gravimetric, electrical, and optical techniques to relate the quantity of adsorbed water to changes in optical constants, electrical resistance, and capacitance of the film under changing RH conditions. We use several isotherm models to examine the kinetics of water adsorption, which reveals a complex adsorption mechanism that involves at least two different adsorption sites, including a dominant process which is active in the entire measurement range (~0–95% RH) and one which becomes active for RH > 60%. Combining gravimetric and optical techniques, we demonstrate the possibility of humidity sensing in the 0–95% RH range with ±2.5% resolution.

## Methods

Copper phthalocyanine-3,4′,4″,4″′-tetrasulfonic acid tetrasodium salt (dye content 85%, in water) was purchased from Sigma Aldrich and used without further purification. CuPcTs films were deposited from solution on pre-cleaned 5 MHz AT-cut gold-coated quartz crystal microbalance (QCM) crystals with 1 cm^2^ active electrode area, conductive n-doped SiO_2_ substrates, and undoped SiO_2_ substrates with pre-patterned gold interdigitated electrodes (10 nm thickness, 2 μm spacing, 100 digits per electrode). Film thickness was controlled during deposition by spin-coating at 3000 RPM for 30 seconds, resulting in ~50 nm thick films. Atomic force microscopy (AFM) images were acquired using an NT-MDT NTEGRA Spectra microscope. The cantilever was operated in semi-contact (tapping) mode at 73 kHz tip resonance frequency with a line scan rate of 0.7 Hz.

Prior to H_2_O adsorption measurements, films were placed under 10^−3^ Torr (1.33 × 10^−3^ mbar) vacuum for 24 hours. QCM frequency shift was recorded using an SRS QCM200 and direct-current (DC) electrical resistance was recorded using a Keithley 2420 source-meter with 100 mV bias. Frequency shift of the QCM crystals was converted to mass change using the well-known Sauerbrey equation^25^. Electrochemical impedance spectroscopy (EIS) was performed using a Zahner IM6 electrochemical workstation. Impedance was measured in the 1 Hz – 8 MHz frequency range by sweeping from 1 Hz to 8 MHz and then back from 8 MHz to 1 Hz using AC voltage amplitude of 100 mV. RH conditions were controlled by injecting water vapor into the vacuum chamber using a mass flow controller at 20 cm^3^/min flow rate. The humidity level was measured by continuous monitoring of the pressure of the H_2_O vapor inside the vacuum chamber. Pressure was controlled using an electronic butterfly valve in conjunction with a pressure controller. A LabVIEW program was used to control the H_2_O flow and record resistivity of the film and frequency shift of the QCM during the experiment. All measurements were performed at 27 ± 0.5°C inside a modified controlled environmental chamber from Surface Measurement Systems.

Spectroscopic ellipsometry (SPE) measurements were obtained at 1.5 nm steps from 275–1000 nm at 75° incident angle using a J.A. Woollam Co. M-2000 ellipsometer. A flow cell with optical windows was used to control the sample environment during SPE measurements. RH levels inside the flow cell were controlled using an L&C Science RH-200 humidity generator and measured with an additional humidity probe at the outlet of the flow cell. Before initiating SPE measurements, the CuPcTs film was exposed to 3% RH for 3 hours. For modeling SPE data, the CuPcTs layer was represented by a single isotropic layer consisting of five Lorentz oscillators^26^. A Levenberg–Marquardt algorithm was used for fitting oscillator parameters. The fitted region was reduced to the 450–900 nm range to reduce the standard error.

### Data availability

The datasets generated during and/or analyzed during the current study are available from the corresponding author on reasonable request.

## Results and Discussion

### Film morphology

**Figure 1:**
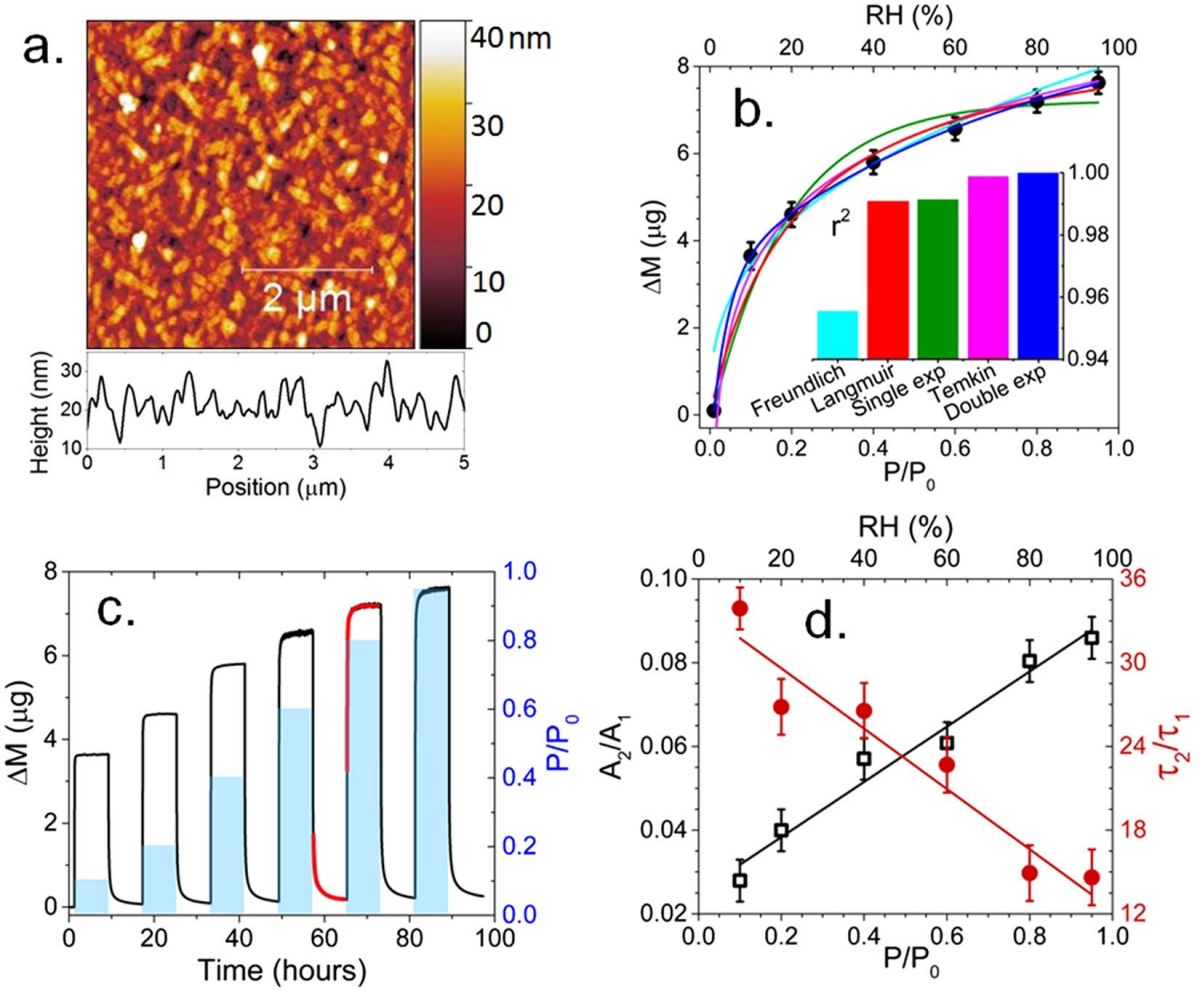
Humidity effect on gravimetric properties. (**a**) AFM image of CuPcTs film. Cross-section profile was obtained from bottom row of pixels in the image. (**b**) H_2_O adsorption isotherm (black circles) fit with five different isotherm models. The r^2^ values of each fit are shown in the inset bar graph. Fit lines correspond to colors used in the bar graph. Error bars correspond to noise in the measured QCM signal. (**c**) Mass change (ΔM) (black, left axis) of CuPcTs film during exposure to pulses of H_2_O vapor (blue, right axis). Red lines indicate examples of double exponential fits. (**d**) Ratio of double exponential fit parameters A_2_/A_1_ (black squares, left axis) and τ_2_/τ_1_ (red circles, right axis). Lines show linear fits. Error bars are propagated double exponential fitting errors.

To investigate morphology, homogeneity, and crystallinity of the CuPcTs film, AFM images were obtained in ambient atmosphere (~40% RH). The CuPcTs film consists of elongated grains up to 600 nm long and 250 nm wide with RMS roughness of 6 nm over a 5 µm length (Fig. 1a). As calculated from SPE measurements, the optical film thickness was roughly 50 nm when measured at ambient RH. The average height visible in the AFM image is ~21 nm, which suggests that pinholes visible in the image penetrate to a depth of at least half of the film thickness. The presence of pinholes and large (10–100 nm diameter) interstitial spaces between grains result in high film porosity and surface area which allows for rapid H_2_O diffusion into the bulk film.

### Gravimetric humidity response

**Figure 2:**
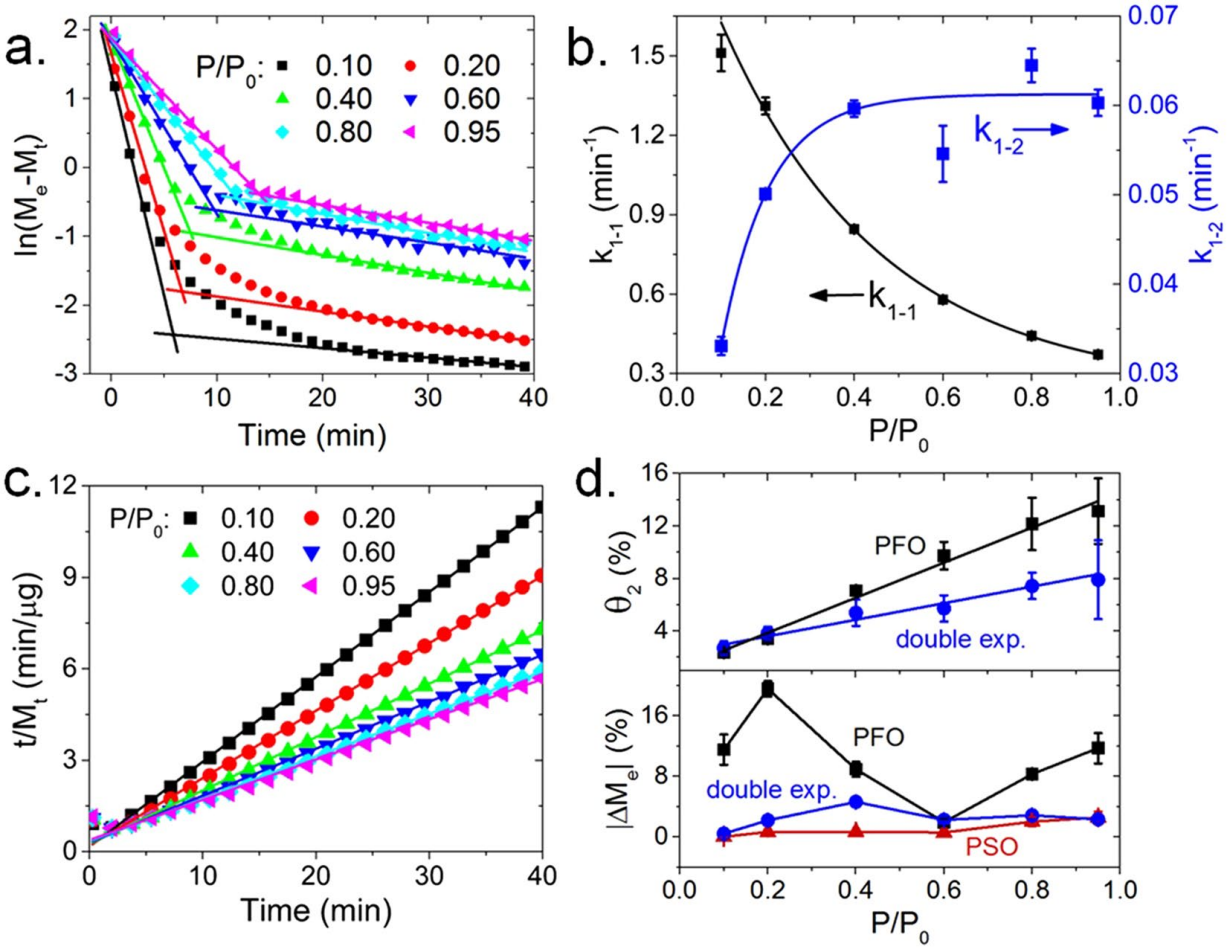
Kinetics modelling. (**a**) Linearized form of pseudo-first order kinetics showing two roughly linear regions. (**b**) Reaction rate constants for first (k_1−1_) and second (k_1−2_) pseudo-first order kinetic processes identified in (**a**), shown with exponential fits. Error bars correspond to linear fitting errors. (**c**) Linearized form of the pseudo-second order kinetics showing highly linear behavior (r^2^ > 0.998). (**d**) Top panel: comparison of *Site*-2 occupancy (${\theta }_{2}$) as estimated from pseudo-first order kinetic and double exponential models. Bottom panel: percent difference (|ΔM_e_|) between measured equilibrium mass (${M}_{e}$) and equilibrium mass predicted by pseudo-first order, pseudo-second order, and double exponential kinetic models. Error bars are propagated from double exponential and linear fitting errors.

**Figure 3:**
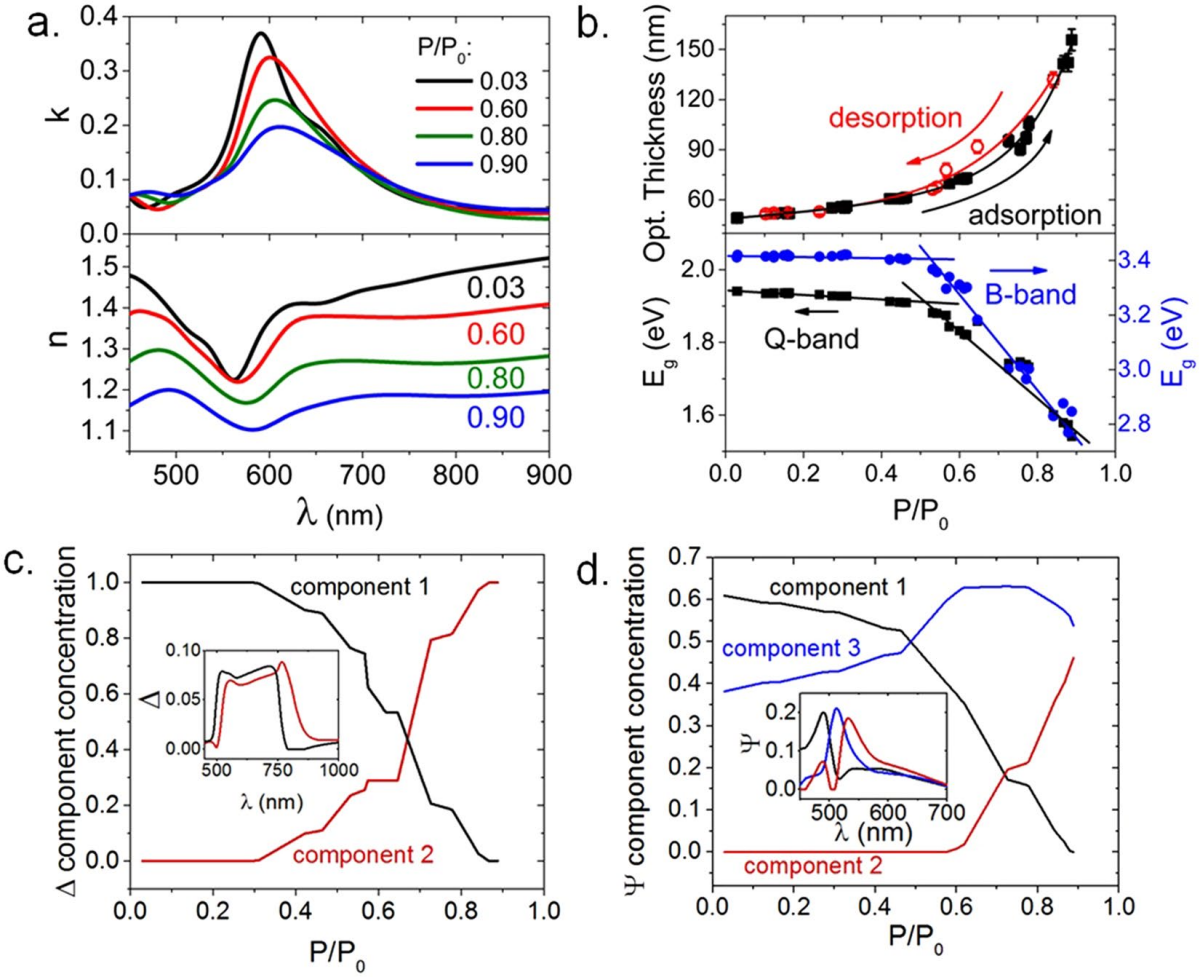
Humidity effect on optical properties. (**a**) Wavelength-dependent extinction coefficient (k) (top panel) and index of refraction (n) (bottom panel) of CuPcTs film under different RH conditions. (**b**) Top panel: Optical film thickness as RH is increased (adsorption, black squares) and decreased (desorption, red circles). Lines show exponential fits. Error bars correspond to errors propagated from fitting to the Lorentz oscillator model. Bottom panel: Bandgap energy (E_g_) of Q-band (left axis) and B-band (right axis). Straight lines show two roughly linear regimes. (**c**) Results from PCA analysis of Δ and (**d**) Ψ obtained from SPE measurements. RH dependence of principal components is shown in main plots with each component’s contribution to the actual spectra shown in the insets. The units of Δ and Ψ are degrees and their principal components are displayed in relative units.

To probe the kinetics H_2_O adsorption on CuPcTs film, as well as quantify mass gain during exposure to different humidity conditions, mass change (ΔM) of the film was measured under different water vapor partial pressures. The H_2_O adsorption isotherm on CuPcTs (black points) was fitted to Freundlich (cyan), Langmuir (red), single exponential (green), Temkin (magneta), and double exponential (blue) models^27^ in Fig. 1b. Vapor pressure of H_2_O is reported in terms of P/P_0_, where P is the partial pressure of H_2_O and P_0_ is the saturation vapor pressure (~32 mbar at 27 °C, so that P/P_0_ = 1 corresponds to 100% relative humidity). The inset bar graph displays r^2^ values for each of the fits. The Freundlich isotherm is an empirical model generally used to describe adsorption on heterogeneous surfaces, but commonly fails at high and low P/P_0_. Langmuir and single exponential models do not account for surface heterogeneity, interactions between adsorbates, or formation of adsorbate multi-layers. Temkin and double exponential models achieve r^2^ > 0.998 and best represent the measured H_2_O adsorption isotherm. The Temkin model is particularly well-suited for predicting gas phase equilibrium with surfaces that contain a distribution of binding energies and explicitly accounts for adsorbate-adsorbate interactions by acknowledging that the heat of adsorption decreases linearly with adsorbate coverage^27, 28^. Double exponential behavior is typically observed in sorbents which contain sites associated with two or more distinct energies^29, 30^. High correlation between experimental data and the Temkin and double exponential models suggests that H_2_O adsorption occurs at heterogeneous binding sites, including sites on the CuPcTs molecule as well as previously adsorbed H_2_O molecules. Results from modelling of adsorption kinetics (Fig. 2) and principal component analysis (Fig. 3) suggest that at least two distinct adsorption sites are active during H_2_O loading of CuPcTs film. Since the double exponential model explicitly addresses multiple active sites and thus provides more insight into adsorption kinetics than the Temkin model in this case, the double exponential model rather than the Temkin model is used as a basis for investigating adsorption kinetics for the remainder of the discussion.

Figure 1c shows mass change (ΔM) of the CuPcTs film during adsorption and desorption of water at different H_2_O vapor pressures (blue pulses). Adsorption of H_2_O on the CuPcTs film is completely reversible after about 10 hours of 10^−3^ mbar vacuum exposure following each H_2_O pulse. Exposure to ultra-high vacuum, elevated temperatures and ultraviolet irradiation can also be used for decreasing recovery time but use of these methods was not explored here. Kinetics of H_2_O adsorption visibly change as P/P_0_ increases; i.e. the rising edge of the mass change becomes more rounded at higher pressures. The ΔM kinetics during both adsorption and desorption of water closely follow double exponential behavior (r^2^ > 0.99) in the 10–95% RH range, as shown by the red double exponential fits to ΔM in Fig. 1c. Exponential heterogeneous (multi-site) adsorption kinetic models are of the form $\,{\rm{\Delta }}M={M}_{{\rm{0}}}+{\sum }_{i=1}^{n}{A}_{i}{{\rm{e}}}^{-t/{{\rm{\tau }}}_{i}}$, where $n$ is the number of active sites, ${A}_{i}$ corresponds to the relative amount of mass adsorbed at *Site*-*i*, and ${{\rm{\tau }}}_{i}\,\,$is the inverse of the adsorption rate constant at *Site*-*i*. Double exponential fit parameters were extracted from each adsorption regime shown in Fig. 1c. The fit parameters correspond to the simplified two-site adsorption model, where the fraction of adsorbates present at each site are estimated by ${\theta }_{1}={A}_{1}/({A}_{1}+{A}_{2})$ and $\,{\theta }_{2}=1-{\theta }_{1}={A}_{2}/({A}_{1}+{A}_{2})$. Values of $\,\theta$,$\,A$, and ${\rm{\tau }}$ are shown in Figures S1 and S2. To examine relative activity of two absorption sites with RH, we used the ratio of pre-exponential factors (A_2_/A_1_) and time constant parameters (τ_2_/τ_1_) derived from double exponential fits shown in Fig. 1d. The ratio of A_2_/A_1_ corresponds to the relative activity of adsorbates at site 2 relative to site 1, while τ_2_/τ_1_ corresponds to timescale associated with the adsorption rate at site 2 relative to the adsorption rate at site 1. At 10% RH, 97.5% of H_2_O molecules adsorb rapidly at *Site*-1 (${{\rm{\tau }}}_{1}$ < 2 minutes) while 2.5% adsorb at *Site*-2 over the course of an hour (${{\rm{\tau }}}_{2}\,$≈ 50 minutes). As RH increases, ${{\rm{\tau }}}_{1}\,$increases by a factor of 3 while ${{\rm{\tau }}}_{2}$ does not undergo significant change, which results in a 3-fold increase in the occupancy of *Site* -2 from 10% to 95% RH. The results strongly suggest that adsorption/desorption of water can be described by a double active site model that includes one strongly hydrophilic site which becomes quickly saturated as RH increases (*Site*-1) and one site which becomes more active as *Site*-1 is filled (*Site*-2)^31^. The hydrophilic site is associated with the Na^+^SO_3_^−^ group, since this is the primary structure responsible for hydrophilicity/solubility of CuPcTs. Water saturation of the sulfonyl groups leads to dissociation of Na^+^ ions from SO_3_^−^^12, 15, 16^ and facilitates hydrogen bonding between water molecules until H_2_O clusters merge, resulting in a multilayered meniscus containing mobile Na^+^ ions on the surfaces of CuPcTs domains. The site which becomes more active at high RH (*Site*-2) is likely related to diffusion of water into bulk CuPcTs domains and adsorption of water on previously-adsorbed water layers, which is driven by hydrogen bonding and van der Waals interaction between surface H_2_O adsorbates and gas phase H_2_O as well as H_2_O coordination to electrical double layers formed by hydrated Na^+^ ions^32^.

### Modelling of adsorption kinetics

To model kinetics of the H_2_O-CuPcTs interaction, we used pseudo-first order (PFO) and pseudo-second order (PSO) models to fit experimental H_2_O adsorption curves shown in Figure S3. Single-site H_2_O adsorption is modelled by the rate equation $\,d{M}_{ad}/dt=\,{k}_{2}^{^{\prime} }[{{\rm{H}}}_{2}{{\rm{O}}}_{vap}][{M}_{e}-{M}_{t}]$, where $d{M}_{ad}/dt$ is the rate of mass adsorption, ${k}_{2}^{^{\prime} }$ is the second order rate constant, $[{{\rm{H}}}_{2}{{\rm{O}}}_{vap}]$ is the concentration of H_2_O vapor, and $[{M}_{e}-{M}_{t}]$ is the concentration of active adsorption sites (${M}_{e}$ is the mass adsorbed at equilibrium at a given P/P_0_ and ${M}_{t}$ is the mass adsorbed at time *t* at a given P/P_0_). Under constant humidity conditions, $[{{\rm{H}}}_{2}{{\rm{O}}}_{ad}]\,\,$is constant and can be absorbed into a new rate constant *k*_1_, where $\,{k}_{1}={k}_{2}^{^{\prime} }[{{\rm{H}}}_{2}{{\rm{O}}}_{vap}]$. The PFO rate equation then becomes $\,d{M}_{ad}/dt=\,{k}_{1}[{M}_{e}-{M}_{t}]$. Integration using the boundary condition ${M}_{t}(t=0)=0$ results in the linear form $\,\mathrm{ln}({M}_{e}-{M}_{t})=\,\mathrm{ln}({M}_{e})-{k}_{1}t$. The plot of $\mathrm{ln}({M}_{e}-{M}_{t})$ vs. *t* is shown in Fig. 2a. The value of ${M}_{e}\,$was obtained from the equilibrium/saturation condition of each ΔM signal shown in Figure S3. Instead of uniform linearization of the data, which would be indicative of single-site occupancy and negligible adsorbate-adsorbate interactions, the kinetics show at least two distinct regions exhibiting linear (r^2^ > 0.98) behavior: one region with slope −0.2 to −0.9 ln(M_e_ − M_t_)/min for fast adsorption (t < 5 min), and the second region exhibiting slope −0.020 to −0.25 ln(M_e_ − M_t_)/min for slower adsorption (t > 15 min). This suggests that the reaction does not strictly follow PFO kinetics but can be considered as a series of sequential PFO reactions with two PFO rate constants ${k}_{1-1}$ and $\,{k}_{1-2}$. Figure 2b shows ${k}_{1-1}$ (left axis) and ${k}_{1-2}$ (right axis) plotted as a function of vapor pressure. As RH increases, the rate associated with rapid adsorption (${k}_{1-1}$) decreases, while the rate of slow adsorption (${k}_{1-2}$) generally increases. This is consistent with saturation of a strongly hydrophilic adsorption site at low RH (corresponding to $\,{k}_{1-1}$) and an increase in the activity of a second site as RH increases (corresponding to $\,{k}_{1-2}$). The behavior of ${k}_{1-1}$ and $\,{k}_{1-2}$ resembles the trend in ${{\rm{\tau }}}_{2}/{{\rm{\tau }}}_{1}$ (Fig. 1d). Both ratios ${{\rm{\tau }}}_{2}/{{\rm{\tau }}}_{1}$ and ${k}_{1-2}/{k}_{1-1}$ increase linearly from ~0.03 to ~0.1 as RH increases from 10% to 95%, which suggests that the double exponential behavior modelled in Fig. 1 represents 2 distinct sequentially-occurring PFO reactions. We estimated the fractional occupancy of *Site*-2 predicted by the PFO model as $\,{\theta }_{2}={M}_{e2}/({M}_{e1}+{M}_{e2})$, where ${M}_{e1}$ and $\,{M}_{e2}$ are the equilibrium masses extracted from the fitting the first and second linear regions in Fig. 2a. *Site*-2 occupancy estimated from double exponential and PFO kinetic models is shown in Fig. 2d (bottom panel). Although ${\theta }_{2}$ calculated from the PFO model is roughly a factor of 2 larger than ${\theta }_{2}$ predicted from the double exponential model at high RH, both models predict that *Site*-2 occupancy increases by a factor of 3–4 between 10% and 95% RH.

Results from double exponential and PFO kinetic models suggest that at least 2 distinct sites are active during H_2_O adsorption on CuPcTs. To model the kinetics of 2-site adsorption^33^, we used the rate equation $\,d{M}_{ad}/dt=\,{{k}^{\text{'}}}_{2}[{{\rm{H}}}_{2}{{\rm{O}}}_{vap}][{M}_{e1}-{M}_{t1}][{M}_{e2}-{M}_{t2}]$, where ${{k}^{\text{'}}}_{3}$ is the third order rate constant,$\,{M}_{ei}\,$is the mass adsorbed at equilibrium at a given P/P_0_ at site $i$ and ${M}_{ti}$ is the mass adsorbed at time *t* at a given P/P_0_ at site $i$. Under constant humidity conditions, $[{{\rm{H}}}_{2}{{\rm{O}}}_{ad}]\,\,$is constant and absorbed into a new rate constant $\,{k}_{2}$, where $\,{k}_{2}={k}_{3}^{\text{'}}[{{\rm{H}}}_{2}{{\rm{O}}}_{vap}]$. In the approximation $\,[{M}_{e1}-{M}_{t1}]\approx [{M}_{e2}-{M}_{t2}]$, the pseudo-second order (PSO) rate equation^34^ becomes $d{M}_{ad}/dt=\,{k}_{2}{({M}_{e}-{M}_{t})}^{2}$. After integration using the boundary condition $\,{M}_{t}(t=0)=0$, the linear form of the PSO rate equation is $\,t/{M}_{t}=1/{k}_{2}{{M}_{e}}^{2}+t/{M}_{e}$. Linear fits to the plots of $t/{M}_{t}\,\,$vs. $t$ achieve r^2^ > 0.998 inside the entire measured RH range (Fig. 2c), indicating that the value of ΔM closely follows PSO reaction kinetics due to the presence of 2 different adsorption sites.

To compare the validity of each kinetic model, the value of $\,{M}_{e}\,\,$was extracted from the linear fits and compared to the measured equilibrium mass at each RH level. Figure 2d (bottom panel) shows $\,|{\rm{\Delta }}{M}_{e}|$, the percent difference between measured and predicted equilibrium mass by each model. For the PFO reaction model, we used $\,{M}_{e}=\,{M}_{e1}+{M}_{e2}$. The PSO model predicts the equilibrium mass with <3% error, while the double exponential model achieves <5% error. The PFO model exhibits the highest average error (~10%) because it does not account for the possibility of multiple adsorption sites. The rate constant ${k}_{2}$ was extracted and compared to ${k}_{1-1}$ and ${k}_{1-2}$ (Figure S4). Although the units for first and second order rate constants are different, the PSO rate constant ${k}_{2}$ clearly resembles behavior of the PFO rate constant $\,{k}_{1-1}$, and its value lies between the values of $\,{k}_{1-1}$ and ${k}_{1-2}$ because $\,{k}_{2}$ describes a combination of two sequentially-occurring reactions with rates $\,{k}_{1-1}\,\,$and $\,{k}_{1-2}$. Thus, even while the H_2_O-CuPcTs interaction most closely follows PSO kinetics due to multiple adsorption sites, useful information about the reaction kinetics is obtained by modeling the reaction as a series of sequential PFO reactions. Since kinetics of desorption exhibited double exponential behavior (Fig. 1c) and showed no significant dependence on water vapor pressure, desorption kinetics were not analyzed in terms of PFO and PSO models.

### Optical humidity response

Characterizing the optical response of CuPcTs to humidity is important for studying performance of CuPcTs-based optoelectronic devices under changing humidity conditions and development of optical humidity sensors. Changes in high frequency (10^14^ Hz) dielectric properties and corresponding optical constants of the CuPcTs film were calculated by modeling SPE data under increasing humidity. Figure 3a shows extinction coefficient (k) (top panel) and index of refraction (n) (bottom panel) as a function of wavelength (λ) at different RH levels. Water exposure leads to significant decreases in k between 550 and 650 nm and decreases in n between 450 and 900 nm. Peak positions of the B- and Q-bands shift to lower energies and the probability of π → π^*^ transitions decrease by 50% as RH increases from 3% to 90%. Changes in the slopes of n and k with RH (Figure S5a) indicate the presence of a 2-step process. The values of n (measured at 450 and 650 nm) decrease slowly for RH < 60% and drop sharply at RH > 60%. Similar behavior is observed in the RH dependence of k (Figure S5b). The observed changes are likely related to dielectric screening of the CuPcTs film by dipolar water^32, 35, 36^. At low RH, the film’s dielectric response to H_2_O adsorption is weak, since delocalization of π-electrons in the phthalocyanine ring allows high electronic polarizability and dielectric constant^37^. At RH > 60%, water saturation of CuPcTs domains leads to dielectric screening of the CuPcTs electronic charge carriers by dipolar water layers, which results in dramatic decreases in the values of n and k.

Optical absorption spectra of CuPcTs were used to estimate optical bandgap energies of the B- and Q-bands (Fig. 3b bottom panel) using a Tauc plot (Figure S6). Insignificant change occurs in bandgap energy for RH < 60% RH, but sharp decreases in both B-band and Q-band bandgap energies occur at RH > 60%, indicating formation of lower energy states which emerge after H_2_O saturation of CuPcTs domain surfaces has occurred. This result, related to the behavior of n and k, is caused by dielectric screening of CuPcTs by water. Optical thickness of the CuPcTs film was estimated from SPE data. Exposure to H_2_O vapor increases CuPcTs film thickness by a factor of 3 as RH is increased from 3% to 90% (Fig. 3b top panel). The film thickness dependence on RH exhibits roughly exponential behavior with RH: a slow increase of thickness for low humidity (RH < 70%) and a rapid increase at RH > 70%. The CuPcTs optical thickness change also exhibits slight (~10 nm) hysteresis in the 50–80% RH range between adsorption and desorption processes. The rapid thickness change at RH > 70% is consistent with formation of a mixed H_2_O-CuPcTs phase facilitated by hydrogen bonding between H_2_O adsorbates and gas-phase water molecules^16, 17, 38^. Upon formation of H_2_O multilayers and merging of adjacent adsorbed H_2_O clusters, interactions between mobile Na^+^ ions, CuPc(SO_3_^−^)_4_^15^, and hydrogen-bonded water can facilitate fracture and displacement of solid-phase domains which increases intermolecular spacing and leads to significant film swelling^18, 39–41^.

To investigate the source of dramatic changes that occur in optical constants at RH ≈ 70%, we performed statistical analyses of Δ (phase difference) and Ψ (amplitude ratio) obtained from SPE measurements (original Δ and Ψ spectra are shown in Figure S7). Application of principal component analysis (PCA) revealed that Δ contains 2 major principal components (Fig. 3c). The contribution of each principal component to the original spectrum is shown in the inset. At RH < 30%, the spectrum is a result of a single component. At RH ≈ 30%, a second component appears and its contribution to Δ grows as RH increases. At RH > 70%, component-2 becomes dominant and forms 100% of the contribution to Δ at RH > 90%. Similar behavior is observed in Ψ, which shows contributions from at least 3 components (Fig. 3d). While the concentration of component-3 remains relatively constant throughout the measured RH range, the component-2 fraction increases sharply at RH ≈ 60%. The PCA results support proposed adsorption kinetics models, which indicate that a secondary adsorption process occurs after H_2_O saturation of strongly hydrophilic sites. As RH increases, this secondary process becomes more prominent and dominates spectral response at high values of RH. PCA results indicate that significant changes in optical constants which occur near 60–70% RH are a result of the emergence of a secondary process which becomes active after significant H_2_O adsorption has taken place in the film, which suggests that the secondary principal component is related to dielectric screening caused by H_2_O saturation of CuPcTs domains.

### Electrical humidity response

**Figure 4:**
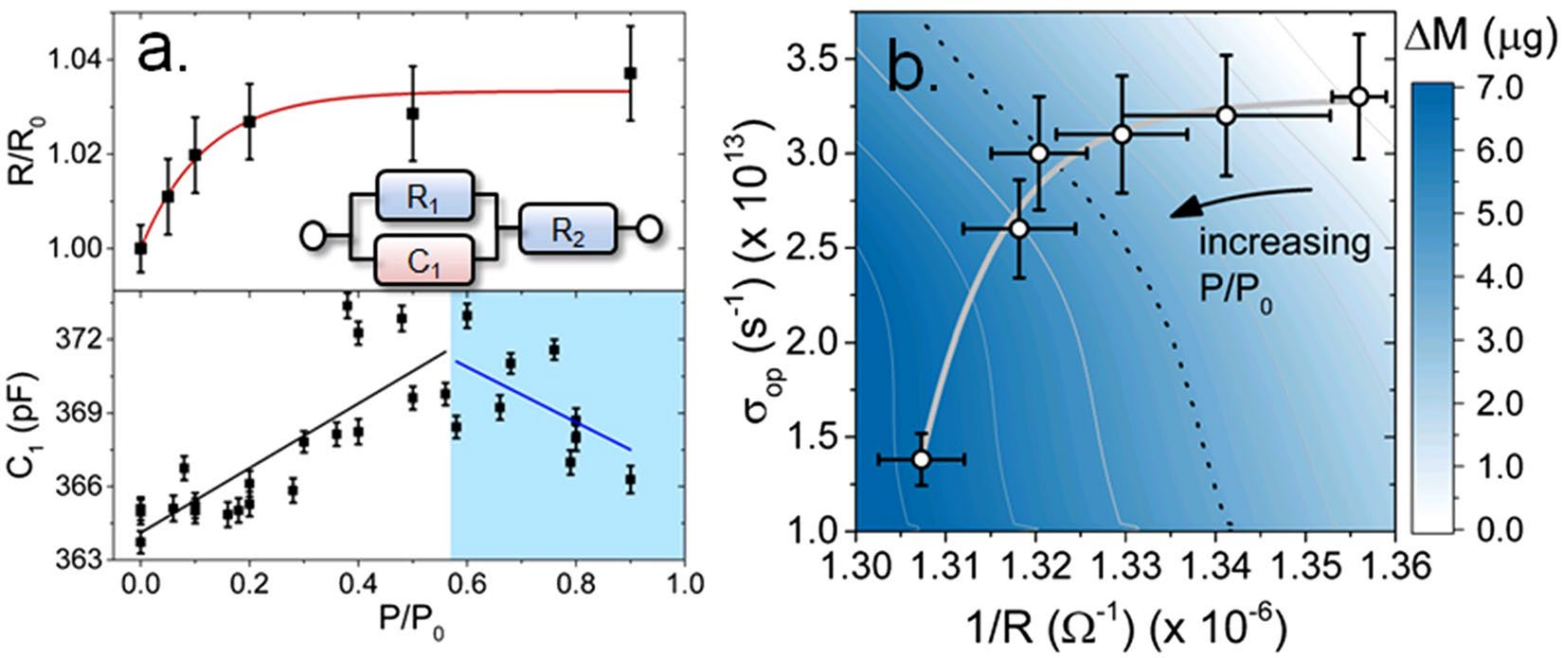
Humidity effect on electrical properties. (**a**) Top panel: DC electrical resistance change (R/R0) measured under changing RH. Red curve is exponential fit. Error bars correspond to uncertainty in R/R_0_ due to low signal to noise ratio. Inset: equivalent circuit used to model EIS data. Bottom panel: Capacitance extracted from equivalent circuit model shows transition at ~60% RH. Error bars are propagated from errors in fitting to equivalent circuit model. (**b**) Relationship between optical conductivity (σ_op_) and electrical conductivity (represented by 1/R, where R is the DC resistance), plotted with mass change (ΔM) of the CuPcTs film during uptake of water and an exponential fit (gray curve). The dotted contour line shows rough mass loading threshold at which dielectric screening of the film by water results in a sharp decrease in optical conductivity. Error bars are propagated from measurements of R/R_0_ and extraction of ε_2_ by fitting to the Lorentz oscillator model.

The most common phthalocyanine-based humidity sensors rely on measurement of direct-current (DC) electrical resistivity measurements. Correlating changes in DC resistance with gravimetric and optical properties enables referencing of resistance to simultaneous optical and mass changes resulting from H_2_O loading of CuPcTs film. Figure 4a shows relative changes in the DC resistance (R/R_0_) as RH is increased. The value of R/R_0_ exhibits roughly exponential behavior, reaching equilibrium at ~50% RH, which suggests that water adsorption alters DC conductivity of the film only before water saturation of CuPcTs domain surfaces has occurred. The adsorption of water onto previously adsorbed H_2_O clusters or multilayers in the mixed H_2_O-CuPcTs phase at RH > 60% does not further affect carrier mobility. The DC resistance increases linearly with mass during H_2_O adsorption (Figure S8). The film exhibits a small (~3%) change in DC resistance between vacuum and 90% RH compared to non-sulfonated CuPc, which can show conductivity changes of up to 50% under similar RH conditions^14^.

To further investigate electrical response of CuPcTs, EIS was performed to study dielectric properties of the CuPcTs film in the 1 Hz – 8 MHz frequency range during exposure to H_2_O. A Nyquist plot of imaginary vs. real impedance (black points, Figure S9) shows a suppressed single semi-circle, suggesting that a modified Randles equivalent circuit model is appropriate for fitting (inset in Fig. 4a)^42^. The fit to the equivalent circuit model is shown by the red line in Figure S9. Fitting was performed on impedance spectra measured from 0% to 90% RH. Both R_1_ and R_2_ undergo slight changes at RH ≈ 57% (Figure S10). The value of R_1_ corresponds to the film resistance and changes insignificantly. The value of R_2_ corresponds to resistance at the film-electrode interface and exhibits a small transition near RH ≈ 57%. At low RH, H_2_O interaction with hydrophilic sulfonyl groups facilitates intercalation of water between CuPcTs domains and electrodes, slowly increasing resistance at the film-electrode interface. At RH > 60%, the formation of H_2_O layers containing mobile Na^+^ ions can increase the effective contact area at electrodes and result in improved electronic transport by allowing charge carriers to hop between hydrated ionic structures consisting of coordinated Na^+^ and dipolar H_2_O, leading to a decrease in R_2_. Film capacitance (C_1_) exhibits a sharp change at RH ≈ 60% (Fig. 4a bottom panel). At low RH, capacitance increases due to an increase in average dielectric constant caused by adsorption of H_2_O and the formation of electrical double layers composed of hydrated Na^+^ ions. At RH > 60%, the value of C_1_ begins decreasing. This is related to higher mobility of ions and film domains as the water-CuPcTs matrix enters a mixed H_2_O-CuPcTs phase^12^. The increased mobility facilitates the breakdown of electrical double layers and a resultant decrease in capacitance. To compare the effect of humidity on electrical and optical conductivities, we extracted optical conductivity (σ_op_) from SPE spectra using the imaginary dielectric constant (ε_2_) by $\,{\sigma }_{{\rm{op}}}={{\rm{\varepsilon }}}_{2}\omega /4\pi$, where ω is the optical frequency. Spectral and RH dependence of ε_2_ is shown in Figure S11. Optical conductivity was calculated at 600 nm (corresponding to the maximum absorbance of Q-band of CuPcTs) and reflects RH dependence at this wavelength. We used 1/R as a measure of electrical conductivity, where R is the DC resistance. Since electrical conductance (1/R) differs from electrical conductivity (*ρ*) by a constant factor related to electrode spacing ($\rho =RA/l$, where *A* is the cross-sectional electrode area and *l* is the channel length between electrodes), the effect of humidity on conductance can be used as an equivalent measure of its effect on conductivity. Figure 4b shows electrical (1/R) and optical (σ_op_) conductivities and relates them to mass change of CuPcTs as the film undergoes water adsorption. The effect of water on electrical conductivity is most pronounced at low RH, when water clustering around hydrophilic moieties hinders electronic charge carrier hopping between adjacent CuPcTs molecules. At ~60% RH (represented by dotted contour line), optical conductivity exhibits a sharp decrease due to the onset of dielectric screening of the CuPcTs domains by a water meniscus and hydrated ionic double layer structures^32, 35, 36^.

**Figure 5:**
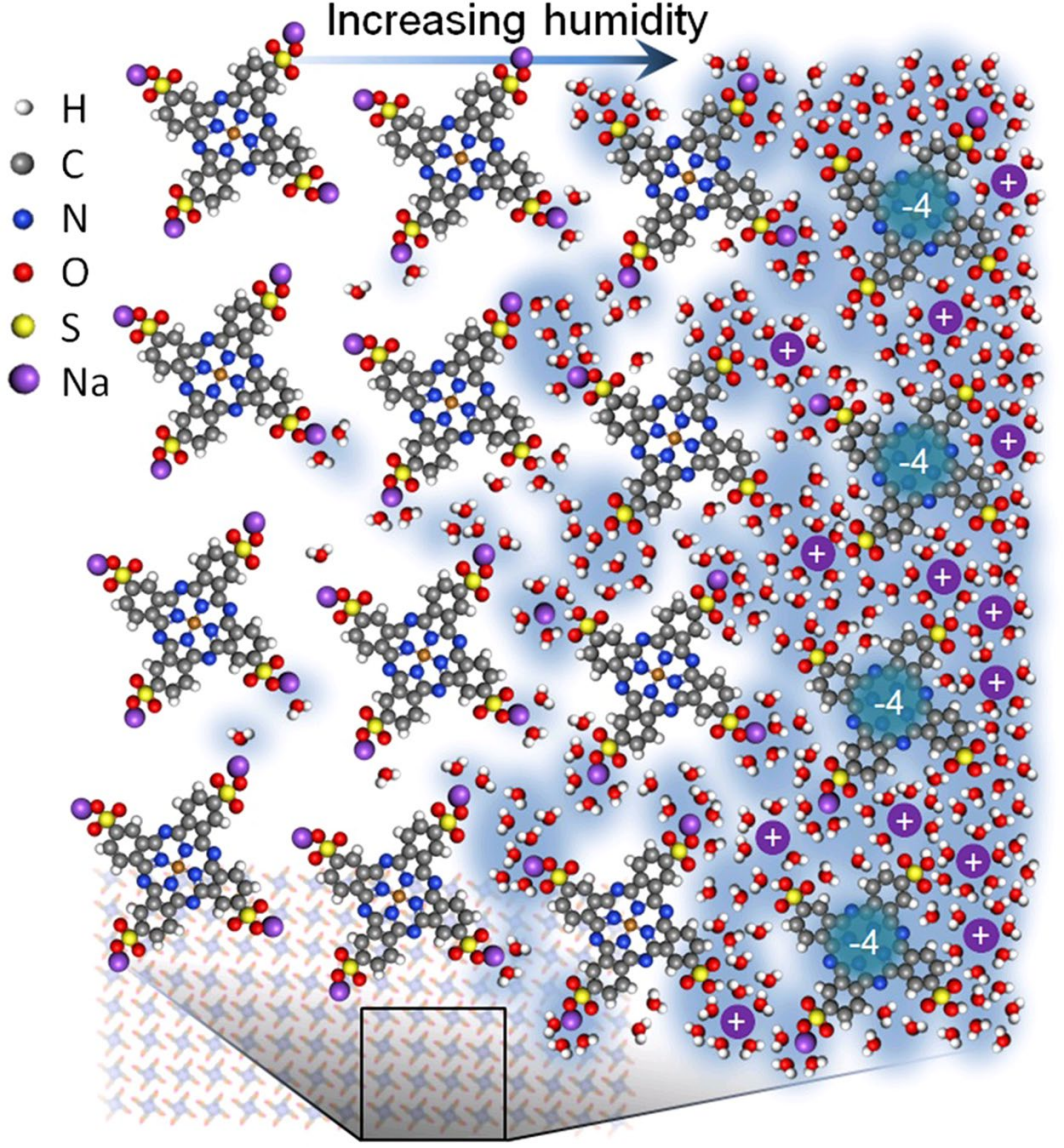
Scheme for water uptake into CuPcTs domain. Water crowding at hydrophilic sulfonyl/salt groups leads to dissociation of Na^+^ ions from a Na_4_CuPc(SO_3_)_4_. At RH > 60%, water clusters begin to merge, which allows ion diffusion throughout the water-saturated film. Mobile Na^+^ ions are represented by purple (+) symbols while CuPc(SO_3_^−^)_4_ ions are left with −4 charges.

The transition in C_1_ occurs in the same RH range (50–70%) at which significant changes in optical constants, bandgap, and film thickness occur. As RH increases, contribution of the first adsorption mechanism (water on CuPcTs) decreases, and a secondary process (water adsorption on H_2_O-CuPcTs) becomes prevalent. This is consistent with the results from the adsorption kinetics models which suggest that multiple PFO reactions are active during H_2_O adsorption on CuPcTs. The combination of these results strongly suggests that H_2_O adsorption on CuPcTs can be described in terms of two sequentially-occurring PFO reaction steps. During the first step, H_2_O molecules undergo rapid adsorption at hydrophilic sulfonyl/salt groups near CuPcTs domain surfaces. Crowding of adsorbates near these groups facilitates salt dissociation leading to formation of 4 Na^+^ ions and negatively charged CuPc(SO_3_^−^)_4_. During the second step (illustrated in Fig. 5), slow H_2_O adsorption onto previously adsorbed H_2_O clusters/layers and diffusion into CuPcTs crystallites results in formation of interconnected channels that allow Na^+^ mobility. Hydrogen bonding between adsorbed water molecules mediated by van der Waals interactions results in formation of a phase which contains mobile H_3_O^+^, Na^+^, and CuPc(SO_3_^−^)_4_ ions. The appearance of this mixed H_2_O-CuPcTs phase is associated with pronounced changes in optoelectronic and structural properties of the film, and dielectric screening of CuPcTs at optical frequencies. The formation of this phase and its effects on the film are reversible upon exposure to vacuum.

### Multi-mode humidity detection sensitivity

**Figure 6:**
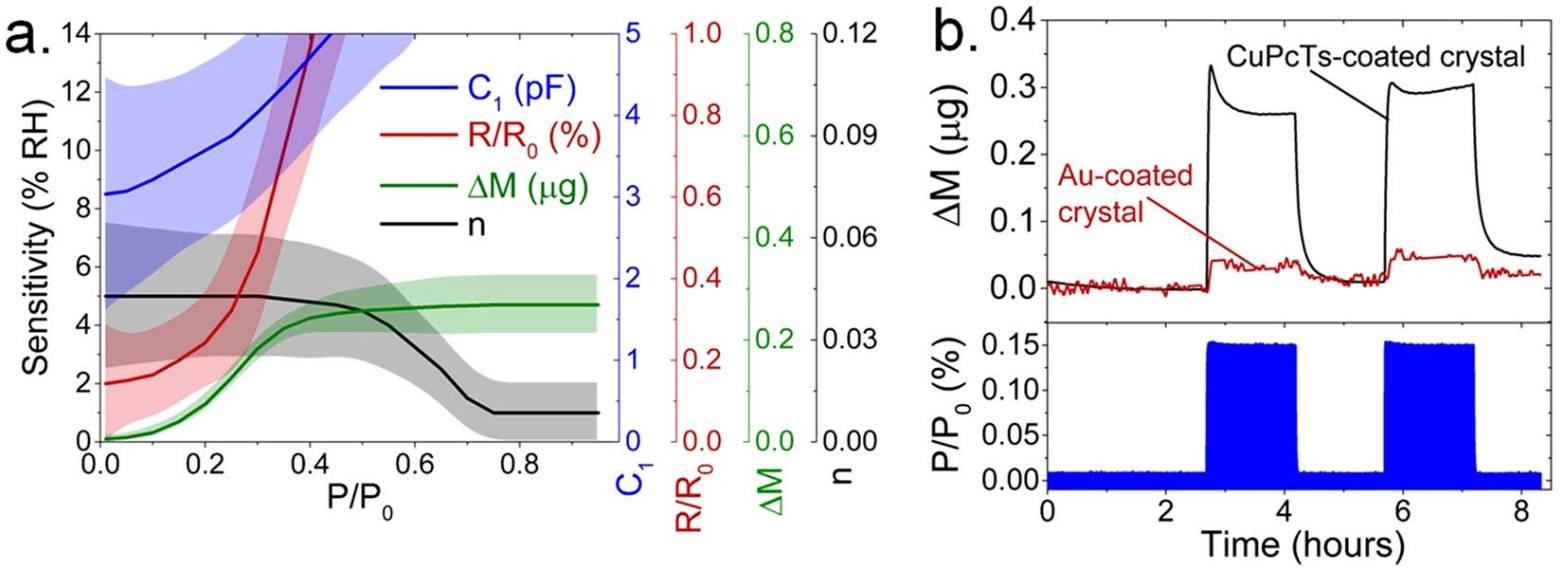
Multimode sensing and detection limits. (**a**) Estimated RH sensitivity achieved by measuring capacitance (C_1_), DC resistivity (R/R_0_), QCM frequency shift (ΔM), and optical constants (n) for RH sensing. Shaded regions represent uncertainty range. Right axes show estimated measurement sensitivity of each technique. Axes units are shown in plot legend. (**b**) Top panel: display of RH sensing limit of mass-based measurements showing ΔM as calculated from frequency shift of CuPcTs-coated QCM crystal (black) and bare gold-coated crystal (red). Bottom panel: Two pulses of H_2_O vapor corresponding to ~0.15% RH which is detected using CuPcTs-coated QCM.

Figure 6a summarizes the results of CuPcTs RH sensitivity as estimated using different measured modes. Here, sensitivity refers to the smallest RH changes detectable by the particular measurement technique at a given water vapor pressure P/P_0_ as estimated from the signal to noise ratio (SNR) for each response. We compare the RH response of capacitance C_1_ (AC impedance spectroscopy in the 1 Hz–8 MHz frequency range), relative resistance change R/R_0_ (DC resistivity), n (optical constant in 300–700 THz range), and ΔM (gravimetric QCM-based measurements). Capacitance response exhibits the lowest sensitivity (~10% RH) at P/P_0_ < 0.2 as a result of low SNR. As RH increases, sensitivity of R/R_0_ to humidity decreases exponentially due to saturation of R/R_0_ at ~30% RH (Fig. 4a). The sharp increase in ΔM which occurs for P/P_0_ < 0.4 (Fig. 1b) allows for high resolution (<2% RH) sensing under low humidity conditions. At RH > 40%, ΔM shows reduced sensitivity of about 4.5% RH due to linear response. This trend is opposite to that of n (optical-mode sensing), which exhibits high RH dependence at P/P_0_ > 0.6 (Figs 3a and S5a) and thus represents the most sensitive (<2% RH) detection mode at high RH. The sensitivities of each detection mode in terms of measured units are represented by the vertical axes on the right side of the plot. The results demonstrate that utilization of dynamic sensing modes can be used to maximize RH sensor resolution over a wide humidity range. In particular, we find that mass-based measurements achieve the highest resolution at RH < 50% while optical techniques achieve the highest resolution at RH > 50%. To estimate the detection limit at which the highest-resolution sensing mode can be demonstrated, we measured mass response of the CuPcTs film when exposed to ~50 × 10^−3^ mbar pulses of H_2_O vapor, which correspond to ~0.15% RH. Figure 6b shows the CuPcTs-coated and bare gold-coated QCM response to 0.15% RH exposure. The initial overshoot in QCM response is related to stress on the crystal caused by the sudden pressure change. The CuPcTs-coated crystal shows sensitivity corresponding to ~0.1% RH. Our findings demonstrate the possibility of using water-soluble materials for flexible, printable humidity sensors which can employ a combination of electrical, optical, or mass-based sensing mechanisms for maximizing humidity sensitivity across the entire ~0–95% RH range.

## Conclusions

We studied the interaction of H_2_O vapor with film of CuPcTs using a multi-modal approach which links gravimetric, broadband dielectric spectroscopy, and DC electrical resistivity measurements. Pseudo-first order, pseudo-second order, and double exponential fits were used to model kinetics of H_2_O adsorption on CuPcTs film. Results indicate that the H_2_O-CuPcTs interaction can be described by two-step pseudo-first order kinetics. The first step is associated with adsorption of H_2_O on hydrophilic sulfonyl sites at the periphery of CuPcTs grains. Adsorption at these sites does not lead to significant changes in optoelectronic properties of the film. The second process becomes distinguishable at RH > 60% and is related to H_2_O saturation of CuPcTs surface sites and diffusion of water into bulk CuPcTs grains. Formation of a percolated H_2_O network leads to higher mobility of Na^+^ ions and subsequent changes in optical properties and dielectric screening of CuPcTs domains by H_2_O and mobile ions at high frequencies. Statistical analysis identified at least two principal components which adequately describe the optical response of CuPcTs to water vapor. Finally, we demonstrate the value of using a multi-modal approach for understanding mechanisms of sensing and referencing optical and electrical changes to the mass of adsorbed water on the surface of the sensor, as well as identification of the most sensitive detection modes for analyte identification. A combination of gravimetric and optical detection modes was selected to enable the highest resolution (±2.5% RH) humidity sensing in the entire 0–95% RH range, with gravimetric detection as the most sensitive mode for RH <50% and optical detection as most sensitive for RH >50%.

## Electronic supplementary material

Supplementary Information
