# Supplementary material for: Multi-mode humidity sensing with water-soluble copper phthalocyanine for increased sensitivity and dynamic range
Source: Sci Rep. 2017 Aug 30;7:9921. doi: 10.1038/s41598-017-10401-2 (PMC5577260; doi:10.1038/s41598-017-10401-2)
Supplement: Supplementary file 1 — Supplementary Information [file 41598_2017_10401_MOESM1_ESM.pdf]

Supporting Information:
Multi-mode humidity sensing with water-soluble copper phthalocyanine for increased sensitivity and dynamic range

Eric S. Muckley[1,2*], Christopher B. Jacobs[1], Keith Vidal[1], Nickolay V. Lavrik[1], Bobby G. Sumpter[1,3], Ilia N. Ivanov[1,2*]

[1]Center for Nanophase Materials Sciences, Oak Ridge National Laboratory, P.O. Box 2008, Oak Ridge, TN 37831-6496
[2]Bredesen Center, University of Tennessee Knoxville, 444 Greve Hall, 821 Volunteer Blvd., Knoxville, TN 37996-3394
[3]Computer Science and Mathematics Division, Oak Ridge National Laboratory, Oak Ridge, TN 37831, USA

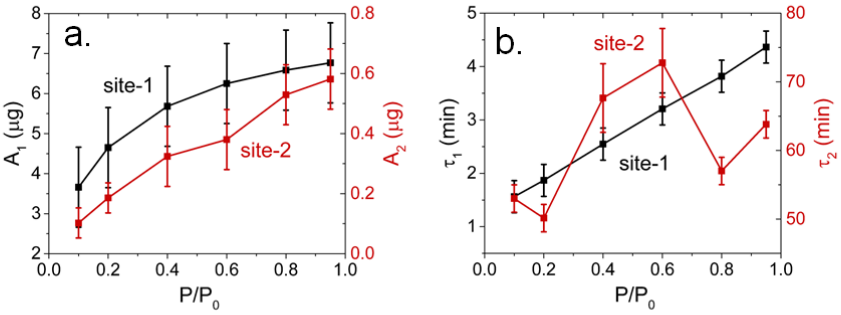

Figure S1. Adsorption site occupancy from double exponential model. (a) Parameters A_1 and A_2 extracted from double exponential fits to ΔM during H_2O adsorption. The values of A_1 and A_2 are associated with the relative amounts of adsorption that occur at two different sites, designated *Site*-1 and *Site*-2, respectively. (b) Parameters τ_1 and τ_2 extracted from double exponential fits. The values of τ_1 and τ_2 are related to the timescales over which H_2O is adsorbed at *Site*-1 and *Site*-2, respectively. Error bars correspond to errors in fitting data to the double exponential model.

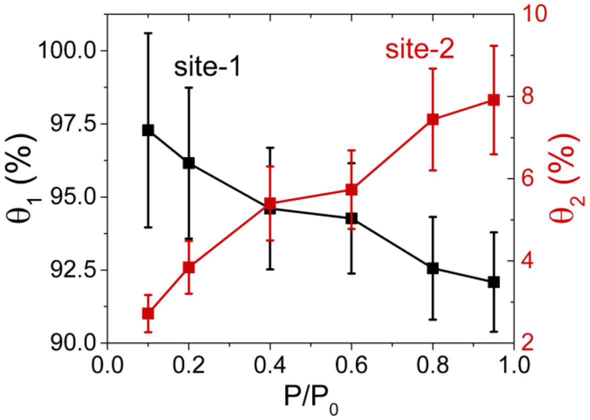

Figure S2. Adsorbate occupancies of *Site*-1 and *Site*-2 (θ_1 and θ_2, respectively) estimated using the two-site double exponential model. Occupancies are calculated from $\theta_1 = A_1/(A_1 + A_2)$ and $\theta_2 = 1 - \theta_2 = A_2/(A_1 + A_2)$. Errors are propagated from errors in fitting to the double exponential model.

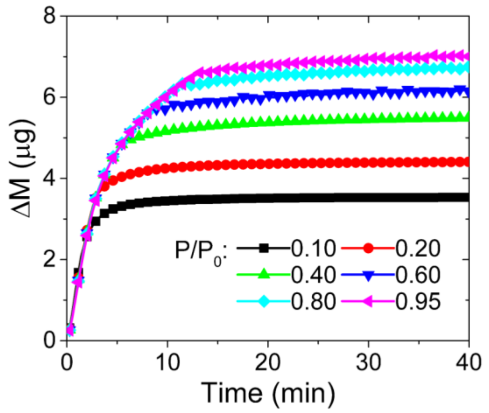

Figure S3. Mass change (ΔM) of CuPcTs film during exposure to different RH levels.

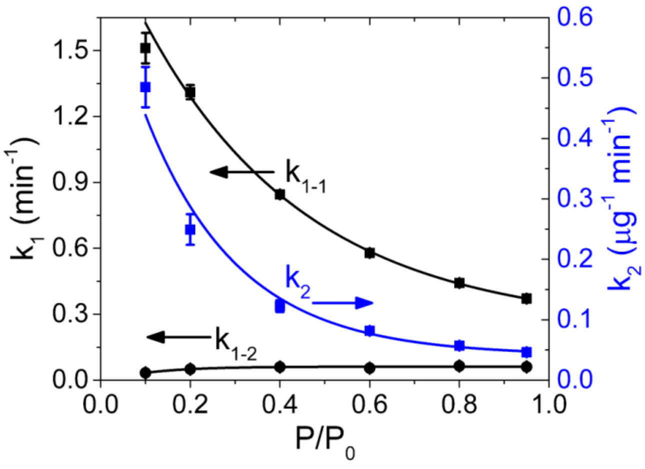

Figure S4. Kinetic rate constants. Constants are extracted from pseudo-first order (k_{1-1} and k_{1-2}) and pseudo-second order (k_2) kinetic models. The behavior of k_2 most closely resembles that of k_{1-1}. Error bars correspond to errors in the pseudo-second order linear fits.

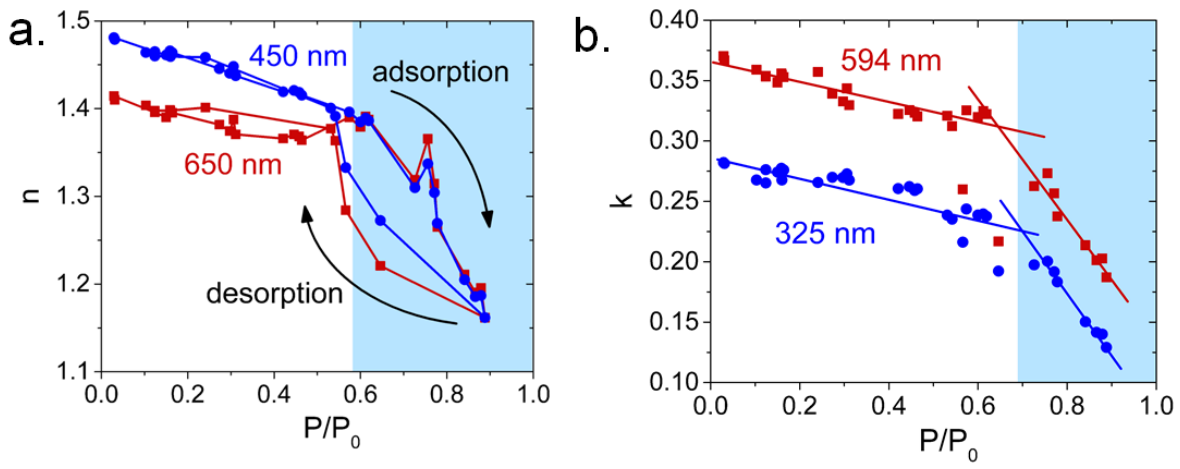

Figure S5. Optical humidity response. (a) RH dependence of index of refraction (n) measured at 450 nm and 650 nm. At RH > 60%, n exhibits a sharp decrease and hysteresis between adsorption and desorption processes is

present. (b) RH dependence extinction coefficient (k) measured at 325 nm and 594 nm. At RH > 70%, k exhibits a sharp decrease.

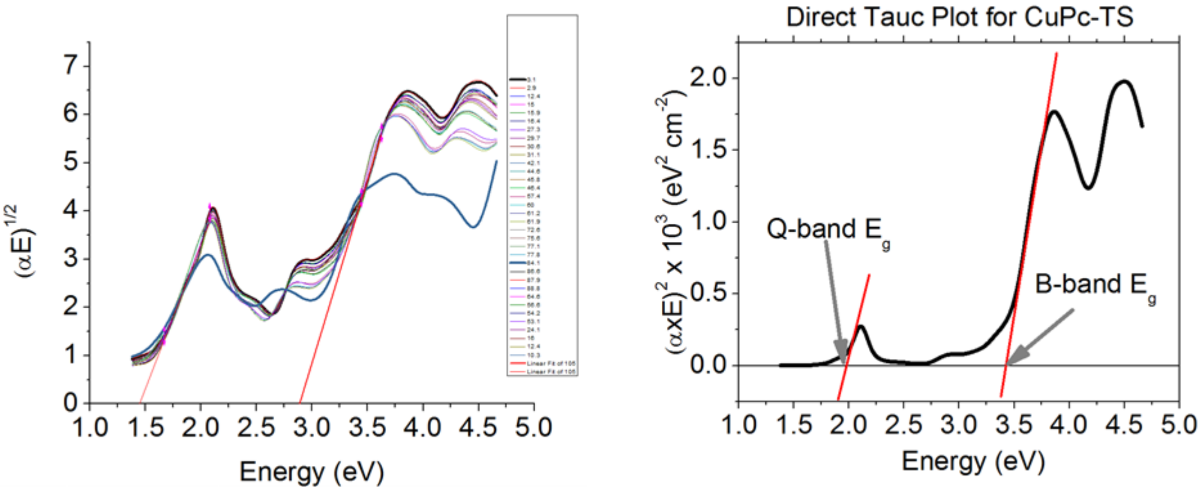

Figure S6. Calculation of bandgap energies. Tauc plots showing estimation of bandgap energy for CuPcTs Q- and B-bands. Left: spectra at different RH levels. Right: detail of bandgap extraction from a single spectrum.

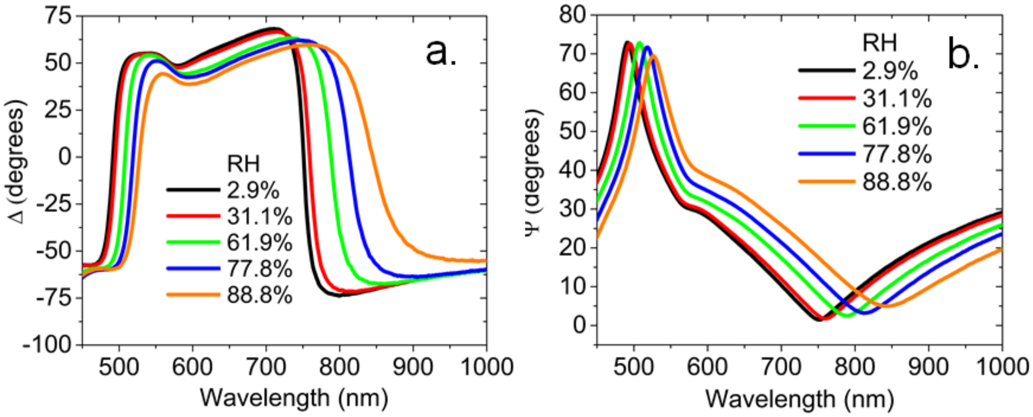

Figure S7. Spectroscopic ellipsometry results. Raw Δ (a) and Ψ (b) SPE spectra measured at different RH levels. The spectra were used to calculate optical constants, film thickness, and principal components.

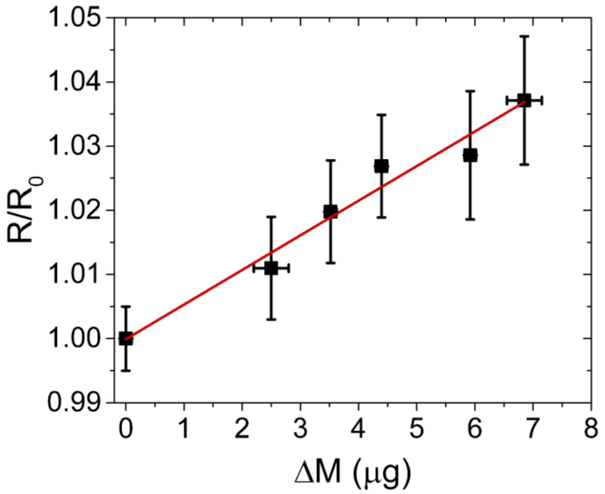

Figure S8. DC electrical resistance response to humidity. DC electrical resistance change (R/R_0) increases linearly with mass of adsorbed H_2O. Error bars correspond to uncertainty in the R/R_0 measurements and noise in the QCM signal.

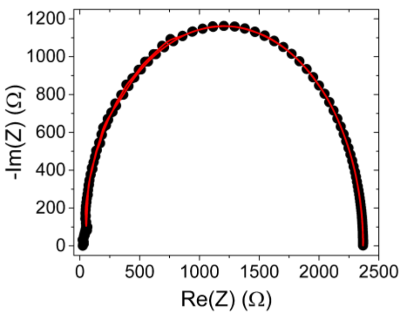

Figure S9. Impedance spectrum. Nyquist plot representation of impedance of CuPcTs film showing imaginary impedance (Im(Z)) vs. real impedance (Re(Z)). Black points are measured data, red line is fit to the equivalent circuit model shown in Figure 4a.

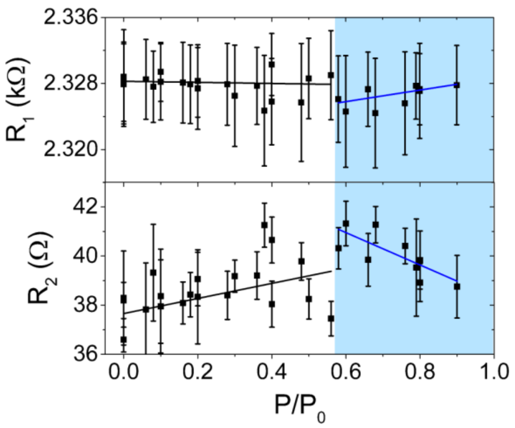

Figure S10. Equivalent circuit parameters calculated from impedance spectroscopy measurements. Resistances R_1 and R_2 extracted from EIS equivalent circuit model. Error bars correspond to error in fitting the impedance data to the equivalent circuit model.

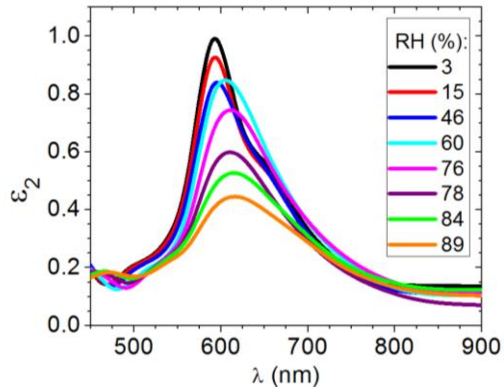

Figure S11. Imaginary dielectric constant. Wavelength and RH-dependence of imaginary dielectric constant extracted from SPE measurements.
